# Prevalence of Psychiatric Disorders among Patients with Granulomatosis with Polyangiitis (Wegener's) and the Predictive Role of Personality Traits

**DOI:** 10.2174/0117450179276345240117043037

**Published:** 2024-01-29

**Authors:** Nazanin Mousavi, Aghil Molaei, Samira Alesaeidi, Nader Eftekhari Seas, Mohammad Effatpanah

**Affiliations:** 1 School of Psychology, Imam Khomeini University, Qazvin, Iran; 2 Faculty of Health, Student Research Committee, Mazandaran University of Medical Sciences, Sari, Iran; 3 Rheumatology Research Center, Tehran University of Medical Sciences, Tehran, Iran; 4 School of Psychology, Kharazmi University, Karaj, Iran; 5 Pediatric Department, School of Medicine, Imam Khomeini Hospital, Tehran University of Medical Science, Tehran, Iran

**Keywords:** Personality traits, Psychiatric disorders, Wegener's disease, Kidneys, Respiratory tract, Patients

## Abstract

**Background:**

Wegener’s disease is an autoimmune condition affecting the respiratory tract and kidneys. Mental health assessment is crucial due to the impact of psychological disorders on the immune system. Despite this, there is limited community-based research on psychiatric disorders and personality traits among patients with Wegener's disease.

**Objective:**

This study aimed to investigate the prevalence of psychiatric disorders and examine the predictive role of personality traits among patients with Wegener's disease.

**Methods:**

A total of 100 patients met the inclusion and exclusion criteria, and all of them were selected to participate in the study. Out of them, 75 individuals completed the questionnaires. The instruments included the SCL-90 questionnaire and the NEO Big Five personality traits. The data were analysed using Stata software, and the prevalence of psychiatric disorders in different patient groups was determined using the chi-square method. The predictive role of personality traits in mental disorders was examined using multivariate regression.

**Results:**

The results revealed that paranoia (53.3%) and depression (44%) had the highest prevalence in terms of psychiatric disorders, while psychosis (17.3%) and hostility (25.33%) had the lowest prevalence. Additionally, the findings demonstrated a positive correlation between most psychiatric disorders and the neuroticism personality trait.

**Conclusion:**

Given the influence of mental disorders on the immune system in Wegener's disease, it is essential to provide psychological care for these patients.

## 1 INTRODUCTION

In 1931, Heintz Klinger, a medical student, first reported Wegener's granulomatosis disease, which encompasses a broad range of clinical manifestations [1]. Frederick Wegener provided a more comprehensive description of the disease and distinguished it from polyarteritis nodosa in 1936 [2]. The disease is characterized by inflammation of the upper and lower respiratory tracts, vasculitis, and glomerulonephritis. The most commonly affected areas include the upper respiratory tract, lungs, kidneys, skin, eyes, ears, orbits, joints, and lymph nodes, in that order of frequency [3]. During the initial stage of the disease, granulomatous mucosal inflammation primarily affects either the upper or lower respiratory tract, accompanied by vasculitis in the acute phase [4]. In the peripheral nervous system, cerebrovascular vasculitis can manifest as polyneuropathy or mononeuritis multiplex. The central nervous system (CNS) exhibits three distinct patterns: 1) vasculitis may affect the small to medium vessels of the brain or spinal cord during the general course of the disease, 2) granulomatous masses originating from the upper respiratory tract can invade adjacent cartilage and bone, causing damage to CNS structures, such as the ocular circuit and meninges in the brain, and 3) granulomatous lesions may develop within the brain tissue, such as in the meninges or brain itself [5].

Wegener's disease, an autoimmune condition with an unknown etiology [6], affects individuals across all age groups but shows a higher prevalence in middle-aged populations, affecting both men and women equally [7]. While comprehensive psychological and psychiatric studies specifically focusing on Wegener's disease patients are lacking, some research has explored the relationship between mental health and autoimmune diseases, such as systemic lupus erythematosus (SLE) [8]. Studies on SLE patients have shown that depressive and anxiety disorders are commonly observed [9]. Depression and anxiety also have a significant impact on heart disease and overall well-being in SLE patients [10]. Factors, such as specific autoantibody profiles, neurological damage, rashes, and cytokine concentrations have been identified as influencers of depression in patients with lupus. Additionally, certain socioeconomic factors, including psychological resilience, contribute to anxiety and depression in SLE patients [11]. High levels of stress, low social support, and psychological stress have negative correlations with the mental and physical health of patients with lupus [12]. Meszaros (2012) reported that more than 39% of patients with lupus experience depression, and over 80% exhibit cognitive dysfunction [13]. Anxiety disorders, cognitive dysfunction, mood disorders, and psychosis are recognized as psychological disorders in patients with lupus, as identified by the American College of Rheumatology [14]. Moreover, hyperactivity, attention deficit, and obsessive-compulsive disorder have been observed in patients with lupus [15]. Previous research has compared psychological disorders between patients with lupus and the general population, demonstrating that depression is four times more prevalent in patients with lupus, while anxiety is twice as common in this group, suggesting that anxiety is a reaction to the disease. Consequently, mental disorders exhibit a high prevalence among autoimmune patients, such as those with lupus. However, these conditions have received comparatively less attention, despite their significant impact on individuals' physical well-being and quality of life. For instance, anxiety and depression can exacerbate cardiovascular diseases, increase the risk of myocardial infarction, suicidal ideation, and mortality, and contribute to decreased quality of life, disability, and loss of employment [16].

Personality traits play a crucial role in understanding mental disorders, leading researchers to explore the relationship between personality and psychopathology. The Five-Factor Model has gained significant attention in classifying personality traits and their relevance to mental disorders. According to various personality trait theories, five major factors have been identified: neuroticism, extraversion, agreeableness, conscientiousness, and openness. These factors have been found to contribute to the development and maintenance of mental disorders [17]. Neuroticism is characterized by traits, such as instability, restlessness, and agitation. Extraversion is associated with boldness, high energy levels, and talkativeness. Openness includes traits related to imagination and intellectual curiosity. Agreeableness is characterized by reliability, cooperative behavior, and good manners. Finally, conscientiousness is associated with traits, such as trustworthiness and a strong sense of responsibility [18]. Several studies have also indicated a potential association between inflammation and personality traits. For instance, low levels of cons- cientiousness have been linked to increased levels of inflammatory markers, including C-reactive protein (CRP) and interleukin-6 (IL-6) [19].

Examining personality and its relationship with disorders enhances our understanding of risk factors, etiology, and therapeutic responses to diseases [20]. Personality serves as a key factor influencing individuals' adaptation and behavior in various environmental circum- stances. In patients with chronic illnesses, emotional states and mood can be significantly impacted [21]. Additionally, it has a significant effect on pain experiences in patients with rheumatoid arthritis [22], and type D personality traits affect patients' quality of life and sleep [23]. Personality traits have the potential to predict the onset of psychiatric disorders [24] and play a substantial role in depression [25]. A study by Giannelou *et al*. demonstrated that extroverted personality traits and anxiety were independently associated with subclinical atherosclerosis in patients with lupus [26]. Research conducted by Gokcen *et al*. demonstrated that type D personality trait is associated with higher levels of anxiety and depression in patients with fibromyalgia [27]. Numerous investigations focusing on personality traits and psychopathology have revealed that psychiatric disorders are characterized by inconsistencies in personality traits [28]. Specifically, depression has been associated with three primary personality traits: high neuroticism, low extraversion, and low conscientiousness [29]. Moreover, personality traits play a crucial role in perceiving situations, events, and mental experiences of pain, influencing the occurrence, severity, and specific domains of psychological disorders [30].

To date, there has been a notable absence of comprehensive psychological investigations exploring the psychological and psychiatric factors in patients diagnosed with Wegener's disease. However, previous studies conducted on other autoimmune and rheumatologic conditions, such as lupus, have indicated a high pre- valence of psychiatric disorders in these patient populations. Moreover, these studies have also highlighted the influential role of personality traits in the development and manifestation of psychological and psychiatric disorders. Building upon these findings, the objective of this study is two-fold: firstly, to determine the prevalence of psychiatric disorders among patients seeking treatment at the Amir Alam Hospital in Tehran, and secondly, to examine and predict the impact of personality traits on psychological and psychiatric disorders in these individuals.

## 2 MATERIALS AND METHODS

### 2.1 Participants

The study sample was comprised of individuals diagnosed with Wegener's disease who were referred to the Amir Alam Hospital in Tehran for treatment. All patients met the diagnostic criteria established by the 1990 American College of Rheumatology (ACR) criteria and the revised Chapel Hill nomenclature for Granulomatosis with Polyangiitis (GPA) [31]. Inclusion criteria encompassed patients who were literate and aged between 20 and 60 years. Exclusion criteria consisted of individuals with acute mental health issues, such as mental retardation. The hospital provided a list of patients with Wegener’s disease, from which we identified individuals who met the inclusion and exclusion criteria. Given the low prevalence of the disease, a total of 100 patients with conditions aligning with the study criteria were identified in the hospital, and all of them were selected to participate. Following a telephone interview, they were encouraged to complete the relevant questionnaires using the Porsline software platform, which was made available online. Out of the 100 patients, a total of 75 participants completed the questionnaires in their entirety.

### 2.2 Instruments

#### 2.2.1 SCL-90 Questionnaire

The questionnaire used in this study assessed nine psychiatric disorders, namely depression, anxiety, somatization, interpersonal sensitivity (feelings of inadequacy), obsessive-compulsiveness, phobic anxiety, hostility, paranoid ideation, and psychoticism. Responses were scored on a 5-point Likert scale ranging from 0 (not at all) to 4 (remarkably). To calculate the questionnaire score, the responses for each subscale were scored and summed. The total score was then divided by the number of questions in the subscale, resulting in the average score for each subscale. The average score ranged from 0 (lowest score) to 4 (highest score). An average score of 1 or higher indicated the presence of a psychiatric disorder. In a previous study, the reliability with Cronbach's alpha was reported to be 0.95 [32].

#### 2.2.2 The NEO's Big Five Personality Traits

The questionnaire used in this study was developed by Costa and McCrae (1989) and consisted of 60 five-choice questions. It utilized a Likert scale for scoring and measuring five dimensions of personality traits, with 12 items for each dimension. The five personality traits assessed by the questionnaire were neuroticism, extraversion, openness to experience, agreeableness, and conscientiousness. A low score on the neuroticism subscale indicates high stability and calmness in one's personality, while a high score indicates instability. A low score on the extraversion subscale suggests introversion and isolation, whereas a high score indicates a tendency for attention-seeking. A low score on the openness to experience subscale indicates a conservative attitude, while a high score reflects a willingness to explore new things. A low score on the agreeableness subscale suggests a tendency for controversy and unreliability, whereas a high score indicates submissive behaviour. Lastly, a low score on the conscientiousness subscale implies carelessness and unreliability, while a high score indicates conscientiousness. The internal consistency of the NEO-FFI big five personality factors questionnaire, as reported by McCrae, ranged from 0.74 to 0.89, with an average of 0.81. In a study conducted in Iran, Cronbach’s alpha coefficient was reported between 0.56 to 0.87 [33].

### 2.3 Ethical Consideration

This study received an ethics code from the ethics committee of the Tehran University of Medical Sciences (IR.TUMS.MEDICINE.REC.1400.946). Before implemen- tation of the research project, informed consent was obtained from each participant.

## 3 RESULTS

We utilized Stata software version 14 for statistical analysis in order to assess the prevalence of psychiatric disorders among different groups based on marital status, gender, and educational background. Furthermore, we examined the prevalence of psychiatric disorders among patients with Wegener’s disease and investigated the predictive role of the big five personality traits in relation to these disorders. The study included a total of 75 patients with Wegener's disease, with an average age of 39.42 ± 10.78. Among these patients, 84% were married, and 70.7% were women. In terms of educational background, 30.7% had primary education, 33.3% had a diploma, 24% had a bachelor's degree, and 12% obtained higher education. Regarding the presence of mental illnesses, 12.2% of the participants reported a history of such disorders (Table **1**).

Table **2** presents the prevalence of psychiatric disorders among the patients. The findings indicate that paranoid disorders (53.3%), depression (44%), somati- zation (42.7%), and obsessive-compulsive and anxiety disorders (37.33%) exhibited the highest prevalence rates. On the other hand, psychotic disorders (17.3%), hostility (25.33%), phobia (29.3%), and interpersonal sensitivity (30.7%) demonstrated the lowest prevalence rates among the study sample.

The results of the multivariate regression analysis revealed several significant findings. The normality of the data was assessed by examining the skewness and kurtosis values. According to the assumption that skewness should fall within the range of +/- 2 and kurtosis within the range of +/- 7, the data in this study were found to be normal [34, 35]. Firstly, somatization was found to be 0.37 units lower in men compared to women (P=0.028), and an increase in neuroticism was associated with higher levels of somatization (P=0.001). Additionally, an increase in neuroticism was found to be associated with higher levels of obsessive-compulsive symptoms (P=0.034).

Regarding sensitivity, a study found that for every one-year increase in age, sensitivity decreased by 0.02 units (P=0.026). Neuroticism and conscientiousness were identi- fied as influential factors in sensitivity (P<0.001), with higher levels of neuroticism and conscientiousness being associated with increased sensitivity. However, agreeableness was found to have a negative effect on sensitivity (P=0.002), indicating that a one-unit increase in agreeableness led to a decrease of 0.05 in sensitivity.

Depression was significantly influenced by neuroticism (P<0.001) and conscientiousness (P=0.024). Specifically, a one-unit increase in neuroticism and conscientiousness was associated with a 0.06 and 0.05 increase in depression levels, respectively.

In terms of anxiety, neuroticism was the only factor that had a significant effect (P=0.008). As neuroticism increased, anxiety levels also increased. Similarly, agreeableness was the only factor found to have an effect on hostility (P=0.022), with the direction of the effect aligning with the nature of hostility.

Regarding phobic anxiety, men exhibited 0.30 units less phobic symptoms compared to women (P=0.045), and neuroticism was found to be a significant factor (P=0.004). Specifically, a one-unit increase in neuroticism led to a 0.04 increase in phobic symptoms.

Neuroticism (P<0.001) and agreeableness (P=0.006) were identified as factors significantly influencing para- noid symptoms. An increase in neuroticism was associated with higher levels of paranoia, while an increase in agreeableness was associated with lower levels of paranoia.

Finally, neuroticism was found to have a significant effect on psychotic symptoms (P=0.004). A one-unit increase in neuroticism resulted in a 0.04 increase in psychotic symptoms (Table **3**).

## 4 DISCUSSION

The higher prevalence of psychiatric disorders observed in patients with Wegener’s disease compared to the general population is consistent with findings from previous studies on individuals with chronic diseases. Mood disorders and anxiety are known to be more prevalent among individuals with chronic conditions compared to healthy individuals in the community. As Wegener's disease is a chronic condition, it is expected that the prevalence of these psychiatric disorders would be elevated in patients suffering from Wegener’s disease, making the study of psychiatric disorders in this population particularly significant. In our study, the most prevalent psychiatric disorders among patients with Wegener’s disease were found to be paranoid disorder, depression, somatization, obsessive-compulsive disorder, anxiety, interpersonal sensitivity, phobic anxiety, hostility, and psychosis, in descending order. The prevalence of these disorders in our sample was higher than the reported prevalence in the general population of Iran based on previous studies [36-38]. This suggests that patients with Wegener’s disease may be at a heightened risk for developing psychiatric disorders compared to the general population. To the best of our knowledge, no previous study has comprehensively assessed the prevalence of psychiatric and psychological disorders, specifically in patients with Wegener’s disease. However, a study conducted by Rula *et al*. (2011) on these patients reported a 22% prevalence of depression disorders, which is consistent with our findings [39]. Studies conducted on other rheumatic diseases, such as lupus, have consistently demonstrated higher rates of depression and anxiety compared to the general population. Individuals with medical illnesses generally experience more psychological distress due to the challenges and impact of the chronic disease on their lives [13, 40]. Chronic diseases like Wegener's disease pose ongoing challenges and disruptions to a person's life. The diagnosis itself can be seen as a significant life challenge, and some patients may struggle to cope with these challenges, leading to an increased vulnerability to developing psychological disorders. The diagnosis of psychiatric comorbidity in granulomatosis with polyangiitis presents some challenges, as the overlap of psychiatric symptoms with the physical manifestations of the autoimmune disease can complicate the identification of distinct mental health issues [41]. Additionally, the variable and evolving nature of psychiatric symptoms in granulomatosis with polyangiitis further complicates diagnostic efforts. Overall, our findings highlight the importance of recognizing and addressing psychiatric disorders in patients with Wegener’s disease. In a clinical context (like a hospital), early identification and appropriate management of these disorders are crucial in providing comprehensive care for individuals with Wegener's disease and improving their overall well-being. Future research should aim to better describe the phenomenology of psychiatric signs and symptoms and continue investigating the specific factors contributing to the development and maintenance of psychiatric disorders in this population and explore effective interventions to support their mental health.

Wegener's disease is an autoimmune condition influenced by various factors, including genetics, hormones, and the environment. Research has demons- trated that mental states, such as mental stress and depression, can contribute to the acceleration of inflam- mation and autoimmune diseases [42]. There is a known association between the immune system and mental disorders, such as anxiety, depression, schizophrenia, and bipolar disorder. These disorders have been linked to the dysregulation of T cell functions and the presence of proinflammatory cytokines, including interleukin-6 (IL-6), IL-2 receptor, IL-1β, IL-17A, and C-reactive protein [43]. T cell function and proinflammatory cytokines play a vital role in the pathogenesis of Wegener's disease [44]. The high prevalence of psychiatric disorders in Wegener's disease can also be attributed to the presence of proinflammatory factors, which are common to both psychiatric disorders and Wegener's disease. These shared factors may contribute to the increased risk and occurrence of psychiatric disorders in individuals with Wegener's disease. Understanding the relationship between psychiatric disorders and autoimmune diseases like Wegener's disease is important, as it sheds light on the complex interplay between the immune system, inflammation, and mental health. Further research is needed to explore the underlying mechanisms connecting these disorders and to identify potential therapeutic targets for managing psychiatric symptoms in individuals with Wegener's disease. By addressing both the physical and mental aspects of the disease, more comprehensive care can be provided to individuals with Wegener's disease, leading to improved overall outcomes and quality of life.

The second aim of this study was to examine the predictive role of personality traits in psychiatric disorders in patients with Wegener's disease. The results indicated that the personality trait of neuroticism positively predicted most psychiatric disorders. Specifically, an increase in neuroticism traits was associated with a higher likelihood of experiencing psychiatric disorders. These findings are consistent with previous research conducted by Malouff *et al*. (2005), which demonstrated a link between clinical disorders and high levels of neuroticism, as well as low levels of conscientiousness, agreeableness, and extraversion. Individuals possessing these personality traits tend to be more susceptible to clinical psychological disorders [45]. Similar results were observed in a study conducted by Habibi *et al*. (2013) on personality traits and mental health in individuals with addiction. Their findings align with our study, highlighting the significant relationship between personality traits and various psychological disorders [46]. Additionally, Koh *et al*. (2015) reported that neuroticism personality traits were associated with higher levels of depression, anxiety, and somatization. Individuals exhibiting high levels of neuroticism were more prone to experiencing anxiety, depression, and somatization symptoms [47]. These consistent findings across studies suggest the importance of considering personality traits, particularly neuroticism, in the understanding and prediction of psychiatric disorders. Recognizing the role of personality traits can contribute to the development of targeted interventions and treatment approaches for individuals with Wegener's disease and other psychiatric conditions. Therefore, further research is needed to explore the underlying mechanisms linking personality traits and psychiatric disorders and to investigate potential interventions that may help mitigate the impact of these disorders on individuals' well-being.

The vulnerability hypothesis suggests that personality traits contribute to a general vulnerability, increasing the likelihood of experiencing multiple psychological dis- orders. Additionally, the pathoplasty model proposes that personality factors influence the course and severity of psychological trauma, although they do not establish a causal relationship [48]. Personality traits have an impact on our perception of events, the environment, and individuals' subjective experience of pain [49]. One specific personality trait discussed in this study is neuroticism, characterized by emotional reactivity and negative responses to threats, frustration, and loss. Neuroticism is associated with irritability, anger, sadness, anxiety, worry, and a propensity for violence. Individuals high in neuroticism tend to exhibit negative and disproportionate reactions to life challenges, such as illness [50]. Overall, neuroticism is associated with deviant behavioral and cognitive patterns and elevated stress levels, which contribute to the development of psycho- logical disorders [48]. Neuroticism is linked to various psychological factors, including anxiety, dep- ression, pessimism, feelings of hopelessness, impulsivity, aggres- sion, low self-esteem, vulnerability to stress, and chronic negative emotions [51]. Neurotic individuals are more prone to experiencing negative emotions, stress, and mental distress [30]. Another personality trait of interest is agreeableness, which encompasses attributes, such as self-sacrifice, cooperation, helpfulness, patience, flexi- bility, generosity, and compassion [52]. Individuals high in agreeableness demonstrate these characteristics more prominently in their personalities. It has been suggested that agreeableness plays a fundamental role in main- taining positive interpersonal relationships and minimizing conflicts. In other words, individuals with high agree- ableness tend to engage in positive behaviors and establish harmonious relationships with others, compared to those with lower levels of agreeableness who may exhibit more provocative behaviors [53]. Several researchers have emphasized the significance of agree- ableness as a fundamental concept in assessing individual differences [54]. Agreeableness plays a motivating role in fostering interpersonal relationships and minimizing conflicts. Individuals high in agreeableness strive to exhibit positive behaviors and cultivate harmonious relationships while minimizing provocative actions towards others [53]. Conversely, individuals with difficulties in social relationships and a tendency towards isolation face an increased risk of mental disorders. This suggests a close association between social relationships and mental health issues [55]. Consequently, individuals with lower levels of agreeableness may encounter challenges in their social interactions, rendering them more susceptible to psychological disorders. Conscien- tiousness, another personality trait, is characterized by attributes, such as responsibility, trustworthiness, and discipline in individuals [56]. Conscientiousness has been found to positively correlate with overall life performance, self-efficacy, and individual achievements. Higher levels of self-efficacy are associated with a lower prevalence of psychiatric disorders [47]. However, the results of this study demonstrated a positive relationship between conscientiousness and certain disorders, such as depression. It is possible that the disease itself and the physical conditions of the patients may have influenced these findings. Therefore, conducting more extensive investigations on the personality traits of patients with Wegener’s disease in future studies is recommended. Wegener’s disease is an autoimmune condition involving the immune system. As mentioned, psychiatric disorders are associated with immune system dysfunction. Consequently, it is advisable to consider psychology and psychiatry interventions alongside physical activities to address the psychological impact of Wegener's disease.

The present study had certain limitations that need to be acknowledged. Firstly, the low prevalence of Wegener's disease posed challenges in accessing an adequate number of patients for the study. Consequently, the sample size was relatively small, which may limit the generalizability of the findings. To overcome this limitation, it is recommended that future studies include samples from multiple provinces or regions to enhance the representativeness of the results and provide a more comprehensive understanding of psychiatric disorders in Wegener's disease.

## CONCLUSION

In conclusion, the findings of this study highlight the increased prevalence of mental disorders among individuals with chronic diseases, such as Wegener's disease. The impact of these diseases on individuals' personal and social lives, coupled with the physical burden they impose, contributes to the elevated rates of mental disorders observed in this population. Among the various psychological factors that influence the development of mental disorders, personality traits play a significant role. Specifically, harmful personality traits like neuroticism are associated with a higher likelihood of experiencing psychological disorders, whereas positive traits, such as extraversion, serve as protective factors against mental disorders. Therefore, it is crucial to identify these personality traits and mental disorders in patients with Wegener's disease in a timely manner and implement necessary interventions to improve their mental well-being. By addressing these factors, healthcare professionals can enhance the overall quality of life for individuals living with chronic diseases and promote better mental health outcomes. Further research and interventions focused on understanding and managing personality traits and mental disorders in this population are warranted.

## Figures and Tables

**Table 1:** Frequency and frequency percentage of people based on demographic information.

**Variables**	**Frequency**	**Percentage**
**Marital status**	-	-
Single	12	16
Married	63	84
**Gender**	-	-
Woman	53	70.7
Man	22	29.3
**Education**	-	-
Primary degree	23	30.7
Diploma degree	25	33.3
Bachelor’s degree	18	24
High-level degree	9	12
**Psychiatric**	-	-
Yes	9	12.2
No	65	87.8

**Table 2:** Prevalence of psychiatric disorders in the research sample group.

Psychiatric Disorder	Without Disorder		With Disorder	
	N	Percentage	N	Percentage
Somatization	43	57.3	32	42.7
Obsessive-compulsives	47	62.67	28	37.33
Interpersonal sensitivity	52	69.3	23	30.7
Depression	42	56	33	44
Anxiety	47	62.7	28	37.3
Hostility	56	74.67	19	25.33
Phobic Anxiety	53	70.7	22	29.3
Paranoid	35	46.7	40	53.3
Psychotic	62	82.7	13	17.3

**Table 3:** The results of multivariate regression analysis to investigate factors affecting psychiatric disorders.

-	**R^2^**	**F**	**P**	**β**	**S.E**	**P**
**Somatization**	0.59	8.06	<0.001	-	-	-
**Marital status**	-	-	-	0.36	0.22	0.109
**Gender**	-	-	-	-0.37	0.17	**0.028**
**Education**	-	-	-	0.03	0.08	0.722
**Psychiatric**	-	-	-	-0.14	0.24	0.565
**Age**	-	-	-	0.01	0.01	0.351
**Neuroticism**	-	-	-	0.05	0.02	**0.001**
**Extraversion**	-	-	-	<0.01	0.01	0.860
**Openness**	-	-	-	<0.01	0.01	0.845
**Agreeableness**	-	-	-	-0.02	0.02	0.170
**Conscientiousness**	-	-	-	0.04	0.02	0.069
**Obsessive**	0.56	7.10	<0.001	-	-	-
**Marital status**	-	-	-	0.17	0.22	0.457
**Gender**	-	-	-	-0.25	0.17	0.142
**Education**	-	-	-	0.05	0.09	0.529
**Psychiatric**	-	-	-	-0.44	0.24	0.075
**Age**	-	-	-	<-0.01	0.01	0.842
**Neuroticism**	-	-	-	0.04	0.02	**0.034**
**Extraversion**	-	-	-	<-0.01	0.01	0.944
**Openness**	-	-	-	0.01	0.02	0.419
**Agreeableness**	-	-	-	-0.03	0.02	0.062
**Conscientiousness**	-	-	-	0.02	0.02	0.437
**Sensitivity**	0.58	7.86	<0.001	-	-	-
**Marital status**	-	-	-	0.32	0.21	0.134
**Gender**	-	-	-	-0.21	0.16	0.191
**Education**	-	-	-	-0.08	0.08	0.330
**Psychiatric**	-	-	-	-0.35	0.23	0.133
**Age**	-	-	-	-0.02	0.01	**0.026**
**Neuroticism**	-	-	-	0.06	0.02	**<0.001**
**Extraversion**	-	-	-	<-0.01	0.01	0.860
**Openness**	-	-	-	0.01	0.01	0.318
**Agreeableness**	-	-	-	-0.05	0.02	**0.002**
**Conscientiousness**	-	-	-	0.08	0.02	**<0.001**
**Depression**	0.57	7.76	<0.001	-	-	-
**Marital status**	-	-	-	0.16	0.22	0.461
**Gender**	-	-	-	-0.20	0.16	0.223
**Education**	-	-	-	-0.05	0.08	0.585
**Psychiatric**	-	-	-	-0.23	0.23	0.323
**Age**	-	-	-	<0.01	0.01	0.625
**Neuroticism**	-	-	-	0.06	0.02	**<0.001**
**Extraversion**	-	-	-	-0.01	0.01	0.330
**Openness**	-	-	-	0.01	0.01	0.404
**Agreeableness**	-	-	-	-0.03	0.02	0.069
**Conscientiousness**	-	-	-	0.05	0.02	**0.024**
**Anxiety**	0.47	5.03	<0.001	-	-	-
**Marital status**	-	-	-	0.19	0.22	0.395
**Gender**	-	-	-	-0.24	0.16	0.150
**Education**	-	-	-	-0.02	0.08	0.803
**Psychiatric**	-	-	-	-0.25	0.23	0.293
**Age**	-	-	-	<-0.01	0.01	0.823
**Neuroticism**	-	-	-	0.04	0.02	**0.008**
**Extraversion**	-	-	-	-0.01	0.01	0.535
**Openness**	-	-	-	0.01	0.01	0.646
**Agreeableness**	-	-	-	-0.03	0.02	0.079
**Conscientiousness**	-	-	-	0.04	0.02	0.054
**Hostility**	0.33	2.89	<0.001	-	-	-
**Marital status**	-	-	-	0.02	0.02	0.924
**Gender**	-	-	-	-0.09	0.15	0.542
**Education**	-	-	-	-0.10	0.08	0.190
**Psychiatric**	-	-	-	-0.10	0.22	0.639
**Age**	-	-	-	-0.01	0.01	0.425
**Neuroticism**	-	-	-	0.02	0.01	0.119
**Extraversion**	-	-	-	-0.01	0.01	0.549
**Openness**	-	-	-	0.02	0.01	0.206
**Agreeableness**	-	-	-	-0.04	0.02	**0.022**
**Conscientiousness**	-	-	-	0.04	0.02	0.069
**Phobic**	0.41	4.05	<0.001	-	-	-
**Marital status**	-	-	-	0.39	0.20	0.053
**Gender**	-	-	-	-0.30	0.15	**0.045**
**Education**	-	-	-	0.04	0.07	0.559
**Psychiatric**	-	-	-	0.20	0.21	0.351
**Age**	-	-	-	<-0.01	0.01	0.718
**Neuroticism**	-	-	-	0.04	0.01	**0.004**
**Extraversion**	-	-	-	0.01	0.01	0.585
**Openness**	-	-	-	-0.01	0.01	0.653
**Agreeableness**	-	-	-	-0.01	0.02	0.447
**Conscientiousness**	-	-	-	0.03	0.02	0.170
**Paranoid**	0.67	11.51	<0.001	-	-	-
**Marital status**	-	-	-	-0.02	0.23	0.940
**Gender**	-	-	-	-0.32	0.17	0.061
**Education**	-	-	-	-0.05	0.09	0.599
**Psychiatric**	-	-	-	-0.06	0.24	0.811
**Age**	-	-	-	-0.01	0.01	0.356
**Neuroticism**	-	-	-	0.06	0.02	**<0.001**
**Extraversion**	-	-	-	0.02	0.01	0.190
**Openness**	-	-	-	<-0.01	0.02	0.906
**Agreeableness**	-	-	-	-0.05	0.02	**0.006**
**Conscientiousness**	-	-	-	0.04	0.02	0.101
**Psychotic**	0.46	4.88	<0.001	-	-	-
**Marital status**	-	-	-	0.19	0.20	0.341
**Gender**	-	-	-	-0.16	0.15	0.279
**Education**	-	-	-	-0.06	0.07	0.417
**Psychiatric**	-	-	-	-0.09	0.21	0.680
**Age**	-	-	-	-0.01	0.01	0.376
**Neuroticism**	-	-	-	0.04	0.01	**0.004**
**Extraversion**	-	-	-	-0.01	0.01	0.510
**Openness**	-	-	-	0.02	0.01	0.230
**Agreeableness**	-	-	-	-0.02	0.02	0.126
**Conscientiousness**	-	-	-	0.04	0.02	0.064

## Data Availability

All data supporting the research findings are available from the corresponding author [M.E] upon request from the editor.

## LIST OF ABBREVIATIONS

CNS: The Central Nervous System
SLE: Systemic Lupus Erythematosus
CRP: C-reactive Protein
IL-6: Interleukin-6
ACR: American College of Rheumatology
GPA: Granulomatosis with Polyangiitis

## References

[1] Andrassy K., Erb A., Koderisch J., Waldherr R., Ritz E. (1991). Wegener’s granulomatosis with renal involvement: Patient survival and correlations between initial renal function, renal histology, therapy and renal outcome.. Clin. Nephrol..

[2] Hoffman G.S. (1994). “Wegener’s granulomatosis”: The path traveled since 1931.. Medicine.

[3] Anderson G., Coles E.T., Crane M., Douglas A.C., Gibbs A.R., Geddes D.M., Peel E.T., Wood J.B. (1992). Wegener’s granuloma. A series of 265 British cases seen between 1975 and 1985. A report by a sub-committee of the British Thoracic Society Research Committee.. Q. J. Med..

[4] Bacon P.A. (2005). The spectrum of Wegener’s granulomatosis and disease relapse.. N. Engl. J. Med..

[5] Drachman D.A. (1963). Neurological complications of Wegener’s granulomatosis.. Arch. Neurol..

[6] Moosig F., Lamprecht P., Gross W.L. (2008). Wegener’s granulomatosis: The current view.. Clin. Rev. Allergy Immunol..

[7] Knight A., Sandin S., Askling J. (2010). Increased risk of autoimmune disease in families with Wegener’s granulomatosis.. J. Rheumatol..

[8] Carvalho J.F., Pereira R.M.R., Shoenfeld Y. (2009). Vaccination, atherosclerosis and systemic lupus erythematosus.. Lupus.

[9] Yilmaz-Oner S., Oner C., Dogukan F.M., Moses T.F., Demir K., Tekayev N., Yilmaz N., Tuglular S., Direskeneli H. (2015). Anxiety and depression predict quality of life in Turkish patients with systemic lupus erythematosus.. Clin. Exp. Rheumatol..

[10] Greco C.M., Li T., Sattar A., Kao A.H., Danchenko N., Edmundowicz D., Sutton-Tyrrell K., Tracy R.P., Kuller L.H., Manzi S. (2012). Association between depression and vascular disease in systemic lupus erythematosus.. J. Rheumatol..

[11] Azizoddin D.R., Zamora-Racaza G., Ormseth S.R., Sumner L.A., Cost C., Ayeroff J.R., Weisman M.H., Nicassio P.M. (2017). Psychological factors that link socioeconomic status to depression/anxiety in patients with systemic lupus erythematosus.. J. Clin. Psychol. Med. Settings.

[12] Dobkin P.L., Fortin P.R., Joseph L., Esdaile J.M., Danoff D.S., Clarke A.E. (1998). Psychosocial contributors to mental and physical health in patients with systemic lupus erythematosus.. Arthritis Rheum..

[13] Meszaros Z.S., Perl A., Faraone S.V. (2012). Psychiatric symptoms in systemic lupus erythematosus: A systematic review.. J. Clin. Psychiatry.

[14] Liang M.H., Corzillius M., Bae S.C., Lew R.A., Fortin P.R., Gordon C. (1999). The american college of rheumatology nomenclature and case definitions for neuropsychiatric lupus syndromes.. Arthritis Rheum..

[15] Slattery M.J., Dubbert B.K., Allen A.J., Leonard H.L., Swedo S.E., Gourley M.F. (2004). Prevalence of obsessive-compulsive disorder in patients with systemic lupus erythematosus.. J. Clin. Psychiatry.

[16] Shen B, Tan W, Feng G, He Y, Liu J, Chen W (2013). The correlations of disease activity, socioeconomic status, quality of life, and depression/anxiety in Chinese patients with systemic lupus erythematosus.. Clin. Developm. Immunol.

[17] Andersen A.M., Bienvenu O.J. (2011). Personality and psychopathology.. Int. Rev. Psychiatry.

[18] McCrae R.R., Costa P.T. (2010). NEO inventories for the NEO personality inventory-3 (NEO-PI-3), NEO five-factor inventory-3 (NEO-FFI-3), NEO personality inventory-revised (NEO PI-R): Professional manual..

[19] Sutin A.R., Terracciano A., Deiana B., Naitza S., Ferrucci L., Uda M., Schlessinger D., Costa P.T. (2010). High neuroticism and low conscientiousness are associated with interleukin-6.. Psychol. Med..

[20] Kotov R., Krueger R.F., Watson D., Achenbach T.M., Althoff R.R., Bagby R.M., Brown T.A., Carpenter W.T., Caspi A., Clark L.A., Eaton N.R., Forbes M.K., Forbush K.T., Goldberg D., Hasin D., Hyman S.E., Ivanova M.Y., Lynam D.R., Markon K., Miller J.D., Moffitt T.E., Morey L.C., Mullins-Sweatt S.N., Ormel J., Patrick C.J., Regier D.A., Rescorla L., Ruggero C.J., Samuel D.B., Sellbom M., Simms L.J., Skodol A.E., Slade T., South S.C., Tackett J.L., Waldman I.D., Waszczuk M.A., Widiger T.A., Wright A.G.C., Zimmerman M. (2017). The Hierarchical Taxonomy of Psychopathology (HiTOP): A dimensional alternative to traditional nosologies.. J. Abnorm. Psychol..

[21] Cameron D.S., Bertenshaw E.J., Sheeran P. (2015). The impact of positive affect on health cognitions and behaviours: A meta-analysis of the experimental evidence.. Health Psychol. Rev..

[22] Donisan T., Bojincă V.C., Dobrin M.A., Bălănescu D.V., Predețeanu D., Bojincă M., Berghea F., Opriș D., Groșeanu L., Borangiu A., Constantinescu C.L., Ionescu R., Bălănescu A.R. (2017). The relationship between disease activity, quality of life, and personality types in rheumatoid arthritis and ankylosing spondylitis patients.. Clin. Rheumatol..

[23] Sariyildiz A., Coskun Benlidayi I., Olmez Engizek S., Deniz V. (2023). The relation of psychological status and type D personality with central sensitization in knee osteoarthritis: Everything is in your mind!. Rheumatol. Int..

[24] Milic V., Grujic M., Barisic J., Marinkovic-Eric J., Duisin D., Cirkovic A., Damjanov N. (2019). Personality, depression and anxiety in primary Sjogren’s syndrome - Association with sociodemographic factors and comorbidity.. PLoS One.

[25] Klein D.N., Kotov R., Bufferd S.J. (2011). Personality and depression: Explanatory models and review of the evidence.. Annu. Rev. Clin. Psychol..

[26] Giannelou M., Tseronis D., Antypa E., Mavragani C.P. (2018). Anxiety and extraversion in lupus-related atherosclerosis.. Front. Psychiatry.

[27] Gokcen N., Coskun Benlidayi I., Tamam L., Demirkol M.E., Yesiloglu C., Guzel R. (2022). Type D personality and self-esteem in patients with fibromyalgia: A cross-sectional case-control study.. Rheumatol. Int..

[28] Krueger R.F., Eaton N.R. (2010). Personality traits and the classification of mental disorders: Toward a more complete integration in DSM-5 and an empirical model of psychopathology.. Pers. Disord..

[29] Kotov R., Gamez W., Schmidt F., Watson D. (2010). Linking “big” personality traits to anxiety, depressive, and substance use disorders: A meta-analysis.. Psychol. Bull..

[30] Bucourt E., Martaillé V., Mulleman D., Goupille P., Joncker-Vannier I., Huttenberger B., Reveillere C., Courtois R. (2017). Comparison of the Big Five personality traits in fibromyalgia and other rheumatic diseases.. Joint Bone Spine.

[31] Pyo JY, Ahn SS, Song JJ, Park Y-B, Lee S-W (2023). Application of the 2022 ACR/EULAR criteria for microscopic polyangiitis to patients with previously diagnosed microscopic polyangiitis.. Clin Exp Rheumatol.

[32] Alavi S.S., Alaghemandan H., Maracy M.R., Jannatifard F., Eslami M., Ferdosi M. (2012). Impact of addiction to internet on a number of psychiatric symptoms in students of Isfahan universities, iran, 2010.. Int. J. Prev. Med..

[33] Mirtaghi GF, Hoshang MA, Mahmood GTs (2001). Application of the NEO PIR test and analytic evaluation of its characteristics and factorial structure among Iranian university students.. Hum Sci. J. Alzahra Univ..

[34] Lomax R., Schumacker R. (2012). A beginner's guide to structural equation modeling..

[35] West SG, Finch JF, Curran PJ (1995). Structural equation models with nonnormal variables: Problems and remedies.. Structural equation modeling: Concepts, issues, and applications..

[36] Mohammadi M.R., Davidian H., Noorbala A., Malekafzali H., Naghavi H., Pouretemad H., Yazdi S., Rahgozar M., Alaghebandrad J., Amini H., Razzaghi E., Mesgarpour B., Soori H., Mohammadi M., Ghanizadeh A. (2005). An epidemiological survey of psychiatric disorders in Iran.. Clin. Pract. Epidemiol. Ment. Health.

[37] Gharraee B., Zahedi Tajrishi K., Sheybani F., Tahmasbi N., Mirzaee M., Farahani H., Naserbakht M. (2019). Prevalence of major depressive disorder in the general population of Iran: A systematic review and meta-analysis.. Med. J. Islam. Repub. Iran.

[38] Mohammadi M.R., Ghanizadeh A., Rahgozar M., Noorbala A.A., Davidian H., Afzali H.M., Naghavi H.R., Yazdi S.A.B., Saberi S.M., Mesgarpour B., Akhondzadeh S., Alaghebandrad J., Tehranidoost M. (2004). Prevalence of obsessive-compulsive disorder in Iran.. BMC Psychiatry.

[39] Hajj-Ali R.A., Wilke W.S., Calabrese L.H., Hoffman G.S., Liu X., Bena J., Clark T., Langford C.A. (2011). Pilot study to assess the frequency of fibromyalgia, depression, and sleep disorders in patients with granulomatosis with polyangiitis (Wegener’s).. Arthritis Care Res..

[40] Tisseverasinghe A., Peschken C., Hitchon C. (2018). Anxiety and mood disorders in systemic lupus erythematosus: Current insights and future directions.. Curr. Rheumatol. Rep..

[41] Jeppesen R., Benros M.E. (2019). Autoimmune diseases and psychotic disorders.. Front. Psychiatry.

[42] Gautam S., Kumar U., Dada R. (2021). Yoga and its impact on chronic inflammatory autoimmune arthritis.. Front. Biosci..

[43] Pape K., Tamouza R., Leboyer M., Zipp F. (2019). Immunoneuropsychiatry - novel perspectives on brain disorders.. Nat. Rev. Neurol..

[44] Spriewald B.M., Witzke O., Wassmuth R., Wenzel R.R., Arnold M.L., Philipp T., Kalden J.R. (2004). Distinct tumour necrosis factor, interferon, interleukin 10, and cytotoxic T cell antigen 4 gene polymorphisms in disease occurrence and end stage renal disease in Wegener’s granulomatosis.. Ann. Rheum. Dis..

[45] Malouff J.M., Thorsteinsson E.B., Schutte N.S. (2005). The relationship between the five-factor model of personality and symptoms of clinical disorders: A meta-analysis.. J. Psychopathol. Behav. Assess..

[46] Habibi Z., Sadeghi H., Haghrangbar F., Madanipour K., Azarnoosh A. (2013). The study of personality characteristics and mental health in addicts.. Procedia Soc. Behav. Sci..

[47] Koh J.S., Ko H.J., Wang S.M., Cho K.J., Kim J.C., Lee S.J., Pae C.U. (2015). The relationship between depression, anxiety, somatization, personality and symptoms of lower urinary tract symptoms suggestive of benign prostatic hyperplasia.. Psychiatry Investig..

[48] Schirmbeck F., Boyette L.L., Valk R., Meijer C., Dingemans P., Van R., de Haan L., Kahn R.S., de Haan L., van Os J., Wiersma D., Bruggeman R., Cahn W., Meijer C., Myin-Germeys I. (2015). Relevance of Five-Factor Model personality traits for obsessive-compulsive symptoms in patients with psychotic disorders and their un-affected siblings.. Psychiatry Res..

[49] Bond M.R. (1984). Pain, its nature, analysis, and treatment..

[50] Costa P.T., McCrae R.R. (1992). Four ways five factors are basic.. Pers. Individ. Dif..

[51] Schmitt D.P., Allik J., McCrae R.R., Benet-Martínez V. (2007). The geographic distribution of Big Five personality traits: Patterns and profiles of human self-description across 56 nations.. J. Cross Cult. Psychol..

[52] Digman J.M. (1990). Personality structure: Emergence of the five-factor model.. Annu. Rev. Psychol..

[53] Gleason K.A., Jensen-Campbell L.A., South Richardson D. (2004). Agreeableness as a predictor of aggression in adolescence. Aggressive Behavior.. J. Int. Soci.r Res. Aggress..

[54] Havill V.L., Besevegis E., Mouroussaki S. (1998). Agreeableness as a diachronic human trait.. Parental descriptions of child personality..

[55] Leong V., Bzdok D., Paulus F.M., Pelphrey K., Redcay E., Schilbach L. (2021). Editorial: Social interaction in neuropsychiatry.. Front. Psychiatry.

[56] Costa P.T., McCrae R.R. (1992). Normal personality assessment in clinical practice: The NEO Personality Inventory.. Psychol. Assess..

